# Role of resilience training on compromising of infertile couples' applicant for divorce: A cross-sectional study

**DOI:** 10.18502/ijrm.v18i3.6717

**Published:** 2020-03-29

**Authors:** Seyed Alireza Afshani, Seyed Mohammad Reza Ghaem Mohammadi, Parisa Khani, Anahita Khosravi

**Affiliations:** ^1^Department of Social Welfare, Social Sciences Faculty, Yazd University, Yazd, Iran.; ^2^Islamic Azad University, Qeshm Branch, Qeshm, Iran.; ^3^Research and Clinical Center for Infertility, Shahid Sadoughi University of Medical Sciences, Yazd, Iran.; ^4^Shahid Sadoughi University of Medical Sciences, Yazd, Iran.

**Keywords:** Resilience, Education, Infertility, Divorce.

## Abstract

**Background:**

Divorce is a social issue, which challenges not only the structure of family but also of a society. Studies have shown that infertility affects the marital boredom. In addition, resilience training and emphasizing on increasing piety (religiousness) can help to decrease this boredom.

**Objective:**

This study aimed to evaluate the resilience training effects on the compromising of infertile couples' applicant for divorce.

**Materials and Methods:**

In this cross-sectional study, 100 infertile couples who had requested for divorce and referred to the Center for consolidation of the family foundation were enrolled. Participants were randomly divided in two categories (n= 50/each): the case group received some consultation classes on social services as well as resilience training by a consultant in 5 sessions lasting 2 hr. In total, 10 hr of treatment; while the control group just received the consultation and social services. Canner and Davidson questionnaires were utilized as pre- and posttest in both groups. Groups answered the resilience's criterion of Canner and Davidson.

**Results:**

The resilience training significantly increased the compromises made by couples in the case group compared to the control (p < 0.01). The results showed that 26% of members of the case group relinquished divorce, while 10% of control group members did the same; this difference was statistically significant (p < 0.01).

**Conclusion:**

The resilience training leads to increased psychological well-being elements and compromises in infertile couples.

## 1. Introduction

Divorce is a social issue which challenges the structure of family and society. Despite all the community and the government efforts for protecting marriage and preventing divorce during different eras, this issue is still an unpreventable fact for some families. As statistics show, it has a rapid growth (1). Infertility is one of the main reasons for divorce among couples, which has many destructive effects on their lives. This issue has sometimes led the couples be encouraged by others to get divorced, remarried or adopt a child (2). Even some studies have actually shown that infertile couples have higher tendencies to get divorced (3). Despite all the scientific and medical advances and the use of novel therapeutic approaches in the treatment of infertility, a significant percentage of infertile couples are not able to treat their infertility. Therefore, the consequences of infertility, no matter personal or social, have affected the infertile couple's lives deeply in all areas (2). For couples failing to have a child, this is not only a disappointing but also a destructive issue (4). They believe that if their infertility is resolved, their relationships with others will improve and they will have more control over their lives (5). One of the important factors that could cause the stability and sustainability of marriages is resilience (6). According to study by Motahari and co-workers, infertility affects the marital boredom, and piety and resilience in infertile women can reduce this boredom in a marriage. Therefore, training on resilience elements and emphasizing on piety can reduce boredom (7). Abbasi and coworkers showed that there is a significant positive relationship between resilience and meta-emotion with psychological well-being infertile women (8). Some studies have considered that compared to the fertile people infertile ones are less resilient (9). Lee and coworkers in their study have shown that infertile women with low levels of resilience can decrease their psychological well-being; moreover, these levels of resilience can also affect so many aspects of life, including the quality of their lives and divorce (10). Grych and co-workers describe resilience to include enhancement in psychological well-being following an adverse experience (11). In addition, in a study by Herrmann *et al*, resilience among infertile couples was considered and it was mentioned that resilience could be notice as a protective factor against infertility-specific distress (12). Moreover, Behzadpour and co-workers concluded that resilience training, along with other components such as self-acceptance, positive relationship with others, autonomy, etc., has been effective in psychological well-being of infertile women (13). Shamsi Sani and Tamannaeifar showed that the quality of life, self-efficacy and resilience level in infertile women is lower than in fertile women (14). In essence, resilience is a process whereby an individual rebounds and grows in a positive direction from stressful life events, whereas recovery implies that an individual just rebounds from a negative experience. Based on these considerations, the following questions were formulated:

• Is there a difference in pre-test and post-test results in two groups (case and control) based on sex?

• Is resilience training correlated with the compromises made by infertile couple's applicant for divorce?

## 2. Materials and Methods

In this cross-sectional study, 100 infertile couples who had requested for divorce from August 2015 to August 2016 referred to the Center for consolidation of the family foundation in Yazd were enrolled. The participants included couples who are literate and diagnosed with infertility and had no prior history of psychological problems or consultation for the same. Couples with a history of psychological problems were excluded.

Participants were randomly divided into two categories (n = 50/each); the case group received some consultation classes on social services as well as resilience training by a consultant in 5 sessions lasting 2 hr. In total, 10 hr of treatment; while the control group just received the consultation and social services; all classes were held at the Family Counseling Center (Mehre Shafa, Yazd). For gathering the data, the Connor-Davidson's resilience scale was used as the pre- and posttest in both groups. Conner and Davidson designed a scale for measuring the resilience that uses a five-point Likert response format. This 25-item scale has ranged from zero (not true at all) to four (true nearly all the time); the total score range is from 0-100, the higher the score, the more the resilience (15). In this study, the Persian version of Connor–Davidson's resilience scale (2003) was used. This scale can easily categorize the resilient and non-resilient ones in the case of clinical and non-clinical groups. It has been normalized by Besharat in Iran (16).

### Description of resilience training sessions

At first, a meeting was held to introduce the resilience model and run the pre-test. The training package was developed for 10 sessions. The contents of this package have been approved by the State Welfare Organization and they were based on the booklets and educational content. The booklet consisted of life-skills education materials that were designed for training classes (17). The couples were trained on the resilience approach for 10 hours as shown in table I. Finally, there was a conclusion offering a summary of all the meetings, discussing the presented topics, and running the post-test

**Table 1:** Resilience approach sssions


**Number of session**	**Content of session**
**Session I (awareness of own abilities)**	A simple definition of self-consciousness and its main components, understanding the strengths and weaknesses of brainstorming, and self-awareness of the objectives
**Session II (improving self-esteem)**	Introducing a clear definition of self-esteem and its components, identifying their own weaknesses and trying to fix them
**Session III (enhancing people's ability to communicate)**	Introducing a simple definition of communication, explaining the appropriate ways of communicating with others, explaining the importance of communication in life
**Session IV (the social connectedness and finding friends)**	A simple definition of the concept of friendship, the role of social support in difficulties, finding the features of appropriate social connectedness
**Session V (goal setting and the way to achieve it)**	Expression of goal, classifying the long-term and short-term goals, setting some short-term and long-term goals and planning to achieve them
**Session VI (Self-efficacy through making decisions)**	Definition of self-efficacy and its effects on life, the definition of the decision and the good decision criteria, making responsible decisions
**Session VII (Self-efficacy through problem-solving)**	An introduction to problem-solving and its steps, thinking about your problems and finding solutions for them
**Session VIII (Self-efficacy through being responsible)**	An introduction to responsibility, expressing the couples' responsibilities in life and the success rate with them, thinking about the reasons of failure in some responsibilities and providing some solutions
**Session IX (anger management, anxiety, and stress)**	Introducing the meaning of anger, anxiety, stress, and their signs, introducing some behavioral methods for controlling anger, relaxation training, and focus on breathing
**Session X (fostering the sense of spirituality and faith)**	Discussing the role of spirituality in life, introducing the meaning of being optimistic and hopeful in life, discussing the role of being optimistic and hopeful in mental health

### Ethical consideration

The study proposal was approved by the Ethical Committee of Research, Shahid Sadoughi University of Medical Sciences, Yazd (code: IR.SSU.REC.1395.108). All volunteers who agreed to participate in this investigation signed a written informed consent.

### Statistical analysis

To determine the validity of this scale, the correlation coefficient of each item was evaluated with the total score, which were between 0.41 and 0.64. In order to determine the coefficient of the scale, the Cronbach's alpha test was used (α = 0.89). Data analysis was performed using the SPSS software, version 24.0 (Statistical Package for the Social Sciences, SPSS Inc., Chicago, Illinois, USA). Chi-square test, independent sample *t* test, and paired sample *t* test were used for statistical analysis. The significance level was defined at p < 0.05.

## 3. Results

The mean ± SD of age in the case and control groups was 32.45 ± 6.06 and 32.10 ± 5.72 yr, respectively. In the case group, the most frequent levels of education were diploma (39%) and the Bachelor of Sciences (17%). However, the illiterate and Ph.D. degree had the lowest frequencies of 4% and 2%, respectively. In the control group, the most frequent levels of education were diploma (45%) and third grade of middle school (16%), and the levels of the illiterate and the postgraduate were 5% and 6%, respectively which were the lowest frequencies. The mean ± SD of the duration of marriage in the case and control groups was 9.4 ± 4.14 and 8.62 ± 3.56 yr, respectively (Table II).

In the case group, the mean ± SD of resilience pre-test in the women and men were 47.14 ± 4.71 and 51.10 ± 5.13, respectively (p = 0.01). These scores in the control group obtained 48.36 ± 7.74 and 49.46 ± 7.61, respectively (p = 0.47).

In addition, in the case group, the mean ± SD of posttest scores of resilience in women and men were 57.86 ± 6.19 and 63.72 ± 6.27 (p < 0.01) and in the control group were 50.34 ± 6.74 and 53/72 ± 6/39, respectively (p < 0.01) (Table III).

Resilience training significantly increased the compromising results (the ratio of resilience to divorce) in the case group compared to the control group (Table II). In addition, resilience training lad to increase the components of psychological well-being in the case group compared to the control group, ultimately leading to increased resilience in them.

Finally, according to table IV, 26% of the members of the case group relinquishment from divorce, while 10% of the members of the control group did the same; hence, this difference was statistically significant (p < 0.01).

**Table 2:** Demographic characteristics of study participants


**Variables**	**Case group (with education)**		**Control group (without education)**	
	**Women**	**Men**	**Women**	**Men**
Age*	29.78 ± 5.25	35.12 ± 5.66	30.08 ± 5.15	34.12 ± 5.60
Educational level**				
**Illiterate**	4 (4)		5 (5)	
**Primary school**	14 (14)		13 (13)	
**Third-Grade middle school**	16 (16)		16 (16)	
**Diploma**	39 (39)		45 (45)	
**Bachelor of science (B.Sc.)**	17 (17)		15 (15)	
**Master of science (M.Sc.)**	8 (8)		6 (6)	
**Doctoral degree (Ph.D.)**	2 (2)		0 (0)	
Duration of marriage* (Yr)	9 ± 4.14		8 ± 3.56	
*Data presented as Mean ± SD **Data presented as n (%)				

**Table 3:** Comparison of mean resilience scores of pre- and posttest among couples


**Exam conditions**	**Case group**				**Control group**			
	**Women**	**Men**	P-value*	**Total**	**Women**	**Men**	P-value*	**Total**
**Pre-exam**	47.14 ± 4.71	51.10 ± 5.13	0.01	49.12	48.36 ± 7.74	49.46 ± 7.61	0.47	48.91
**Post-exam**	57.86 ± 6.19	63.72 ± 6.27	0.01	60.79	50.34 ± 6.74	53.72 ± 6.39	0.01	52.03
P-value**	0.01	0.01	0.01	0.01	0.01	0.01
All data presented as Mean ± SD *Independent sample *t* test **Paired sample *t* test								

**Table 4:** The final decision of the participants in the two groups


**Group**	**Final decision**	
	**Divorce**	**No divorce**
**Case**	74 (0.74)	26 (0.26)
**Control**	90 (0.90)	10 (0.10)
Data presented as n (%). Chi-Square = 8.762, p = 0.01		

## 4. Discussion

Divorce is a fact in today's modern life and is the result of unsolved marital problems and, more importantly, the lack of resilience in couples. Resilience is considered as a kind of self-care behavior in couples that increases the ability to solve problems. This awareness should be developed among couples whose lives do not always meet their expectations, and they may encounter some problems in the family, the workplace, etc. Furthermore, dealing with these conflicts may be short-term and sometimes long-term. This adjustment is an indication of resilience among couples because resilience can be considered as a successful adaptation to adverse conditions such as divorce. There is little research on the effectiveness of resilience training in reducing divorce. Therefore, the present study aimed to address this issue and, to some extent, eliminate the research gap in this field. In order to achieve this goal, the introduction of resilience model and pre and posttest implementation were used. The training package was run for 10 sessions and the contents of the package were approved by the Welfare Organization (17) and, accordingly, the couples were trained on 10 lessons/hr.

The results of the research showed that resilience training significantly increased the compromise among couples in the experimental group compared to the control group. In addition, resilience training has led to increased mental health in the experimental group compared to the control group. The reason for this can be the improvements in capabilities that may have been given more attention to in the resilience training programs. These capabilities included increase in the self-awareness, self-esteem, linkage, goal-orientation, self-efficacy (problem-solving, decision-making, and responsibility), controlling excitements, and meaningfulness.

Overall, our results demonstrated that the resilience training had a significant effect on the compromises made by infertile couples applying for divorce. The results of the present study are in accordance with the study done by Masten, Herrmanne, and colleagues, Yu and colleagues, and Faircloth. In explaining, they concluded that resilience offers a positive outlook on human development, social support, and an unspecific protective factor against infertility distress and quality of life in infertile couples (6, 12, 18, 19).

In addition, we found that training on the components of resilience led to an increase in the components of psychological well-being in these couples. The results of the present study are in accordance with the study done by Liu and colleagues (20), Liu and coworkers (21), Yu and coworkers (18), and Kaboudi and coworkers (22).

According to the results of this study and of the aforementioned previous studies, resilience can predict the psychological well-being in infertile women. Resilience leads to positive changes in the quality of life and psychological well-being (16, 21, 22). Therefore, decrease in the resilience in couples leads to decrease in their psychological well-being. Therefore, by improving resilience, they can accept thoughts, feelings, and events without judging and make some positive changes in their lifestyle, which leads to a positive attitude toward themselves and their lives.

## 5. Conclusion

Resilience can play a significant role in the lives of infertile couples. These families not only have no children, but also some other points, such as turmoil in family and marital relationships, the feeling of being left alone by others, and being blamed by themselves or others, can have an impact on the characteristic and psychological aspects of such couples. According to the results of this study and other similar studies, training on the components of resilience leads to increase in the criterion of psychological well-being in such couples; consequently, it leads to an increase in the compromises made by such couples. Hence, it is recommended to study these kinds of interventions to improve the mental health.

##  Limitation

There are some limitations in this study that should be taken into account in further studies. The participants were chosen from those who referred to Welfare Organization of Yazd City. Moreover, the sample size was small, and for generalizing the results, a larger sample size is required.

##  Conflict of Interest

The authors declare that there is no conflict of interests to report.
